# The Impact of Selection with Diflubenzuron, a Chitin Synthesis Inhibitor, on the Fitness of Two Brazilian *Aedes aegypti* Field Populations

**DOI:** 10.1371/journal.pone.0130719

**Published:** 2015-06-24

**Authors:** Thiago Affonso Belinato, Denise Valle

**Affiliations:** 1 Laboratório de Fisiologia e Controle de Artrópodes Vetores, Instituto Oswaldo Cruz (Fiocruz), Rio de Janeiro (RJ), Brasil; 2 Laboratório de Biologia Molecular de Flavivírus, Instituto Oswaldo Cruz (Fiocruz), Rio de Janeiro (RJ), Brasil; 3 Instituto Nacional de Ciência e Tecnologia em Entomologia Molecular (INCT-EM), Rio de Janeiro (RJ), Brasil; Institut Pasteur, FRANCE

## Abstract

Several *Aedes aegypti* field populations are resistant to neurotoxic insecticides, mainly organophoshates and pyrethroids, which are extensively used as larvicides and adulticides, respectively. Diflubenzuron (DFB), a chitin synthesis inhibitor (CSI), was recently approved for use in drinking water, and is presently employed in Brazil for *Ae*. *aegypti* control, against populations resistant to the organophosphate temephos. However, tests of DFB efficacy against field *Ae*. *aegypti* populations are lacking. In addition, information regarding the dynamics of CSI resistance, and characterization of any potential fitness effects that may arise in conjunction with resistance are essential for new *Ae*. *aegypti* control strategies. Here, the efficacy of DFB was evaluated for two Brazilian *Ae*. *aegypti* populations known to be resistant to both temephos and the pyrethroid deltamethrin. Laboratory selection for DFB resistance was then performed over six or seven generations, using a fixed dose of insecticide that inhibited 80% of adult emergence in the first generation. The selection process was stopped when adult emergence in the diflubenzuron-treated groups was equivalent to that of the control groups, kept without insecticide. Diflubenzuron was effective against the two *Ae*. *aegypti* field populations evaluated, regardless of their resistance level to neurotoxic insecticides. However, only a few generations of DFB selection were sufficient to change the susceptible status of both populations to this compound. Several aspects of mosquito biology were affected in both selected populations, indicating that diflubenzuron resistance acquisition is associated with a fitness cost. We believe that these results can significantly contribute to the design of control strategies involving the use of insect growth regulators.

## Introduction

Mosquitoes are vectors of several human pathogens [1]. *Aedes aegypti*, for example, can transmit yellow fever, dengue and chikungunya viruses [2–5]. This mosquito is specially adapted to living in the urban environment and its distribution has expanded greatly in the last few decades [6]. This expansion has coincided with an increase in the global incidence of dengue, with the disease now affecting around 96 million people annually [7].

In the Americas, Brazil plays an unfortunate leading role in dengue transmisison: in a long-term historical study (1980–2007), our country accounted for more than half of all cases reported across the continent [8]. Critically, the current impact of dengue does not deviate significantly from that scenario [9].

Although in the last fifty years a wide range of studies have focused on the development of a vaccine against dengue virus, the creation of a tool that is effective against all serotypes is still a challenge [10]. Therefore, vector control remains the primary strategy to decrease the risk of dengue, and the use of insecticides is a major component. Currently, pyrethroids (PY) and organophosphates (OP) are the main neurotoxic insecticides used to combat *Ae*. *aegypti* [11,12]. Historical overuse of these compounds resulted in the dissemination of resistance in wild *Ae*. *aegypti* populations, precluding their further utilization in many areas [13–18]. In Brazil for example, several *Ae*. *aegypti* populations exhibit high resistance levels to PY, which has been extensively employed against adults since 2000 [19], and to the OP temephos, a pesticide that has been used for mosquito control since the 1960s [20,21].

Compounds with unrelated mechanisms of action, like insect growth regulators (IGR), are considered a promising alternative for the control of field populations resistant to traditional insecticides [22]. Amongst IGRs, diflubenzuron (DFB) was the first chitin synthesis inhibitor (CSI) employed commercially, initially to control crop pests and flies of veterinary importance [23]. Further studies have also demonstrated the efficacy of DFB against mosquitoes [24–27]. In 2003 the World Health Organization recommended the application of DFB in drinking water, opening up the possibility of its use in *Ae*. *aegypti* control [28]. Recently, DFB was adopted by the Brazilian Dengue Control Program to combat *Ae*. *aegypti* populations, especially those already resistant to temephos [29].

Regardless of the insecticide class, the intense use of a single compound will likely select for resistant individuals. Indeed, resistance is one of the main obstacles facing insecticide-based control programs. However, it is also typically associated with an energetic cost that can negatively influence mosquito biology [30–32]. For this reason, only in environments exposed to continuous, or intense, insecticide applications, will resistant insects exhibit an adaptive advantage over susceptible ones [30]. Consequently, resistance levels are directly related to the frequency of insecticide application, and to the severity of the impact of resistance on mosquito fitness.

Several studies have highlighted the fitness costs associated with insecticide resistance in mosquitoes [33–37]. However, the effects of IGRs are less well studied. In particular, the effects of DFB resistance on *Ae*. *aegypti* have not been evaluated, primarily due to the relative novelty of its use in the field. Therefore, laboratory studies of the dynamics of DFB resistance and any potential fitness effects will prove informative for the design of future CSI-based control strategies. In this work, we exposed two Brazilian field populations of *Ae*. *aegypti* to DFB selection under laboratory conditions for several generations; the evolution of DFB resistance and its impact on mosquito fitness were then investigated.

## Materials and Methods

### Mosquito lines

*Aedes aegypti* eggs were collected with ovitraps set up by Municipal Health Secretaries in the Brazilian cities of Boa Vista (BVT), in the State of Roraima (RR), in 2007, and Aparecida de Goiânia (APG), in the State of Goiás (GO), in 2008. The resulting *Aedes aegypti* adults, obtained in the laboratory, were used to generate the mosquito lines subsequently employed in our experiments. The F2 and F1 generations of BVT and APG, respectively, were used to determine the initial resistance levels and fitness characteristics of each line. Previous work [33] showed that both populations were resistant to temephos, with resistance ratios (RR_95_) of 7.4 (BVT) and 19.2 (APG). Both populations were also resistant to the PY deltamethrin and, genotyping revealed that the kdr Val1016Ile mutation was present at allelic frequencies of 0.067 (BVT) and 0.293 (APG) [33]. The insecticide-susceptible reference line Rockefeller was used as an experimental control [38]. Comparative evaluations of the two Brazilian field populations with Rockefeller strain were performed twice: prior to ('Rock A') [33], and after ('Rock B') the DFB selection process.

### Mosquito rearing

Eggs were allowed to hatch for one hour in a 50 mL plastic cup. Afterwards, approximately 1,000 first instar larvae were carefully transferred to transparent plastic trays (33 X 24 X 8 cm) containing 1 L of dechlorinated water and 1 g cat food (Friskies, Purina, São Paulo/SP). Basins were kept in a Biological Oxygen Demand (BOD) incubator at 26 ± 1°C, during 72 hours, when third instar larvae were collected for use in bioassays [39].

### Diflubenzuron quantitative bioassays

Dose response assays were performed with at least nine concentrations of DFB (PESTANAL; Sigma-Aldrich). Four replicates were used per concentration, each containing 10 third instar larvae in transparent plastic cups filled with 150 mL of DFB solution. One mL of a 2.5% (w/v) solution of grounded cat food was provided once to each cup. Mortality at each developmental stage (larvae, pupae and adults) was checked every two days until all non DFB-exposed control group individuals reached adulthood [40]. The results of these bioassays were used to calculate the doses of DFB that inhibited emergence in 50 (IE_50_), 80 (IE_80_) and 95% of adults (IE_95_), using Probit Analysis [41] and the corresponding resistance ratios (RR), relative to the Rockefeller strain.

### Diflubenzuron selection

Larvae from the two field populations were exposed over successive generations to a fixed concentration of DFB, corresponding to the IE_80_ in the F1 and F2 generations for APG and BVT, respectively (Table 1). Three independent experimental groups of 1,000 third instar larvae were placed in the plastic trays detailed above, with the DFB solution, 1 L of dechlorinated water and 1 g of grounded cat food (Friskies, Purina, São Paulo/SP). For each population, two additional control groups were maintained under the same conditions, but in the absence of DFB. At no point was there an exchange of individuals between any of the groups. Pupae were removed from the basins and transferred to cages each day as they developed. Upon eclosion, adults were fed *ad libitum* with 10% sucrose solution and kept in a temperature and humidity-controlled insectary (26 ± 1°C; 80 ± 10% rh). Females were fed weekly on ketamine and xylazine-anaesthetized guinea pigs to obtain eggs [42]. The BVT and APG populations were exposed to DFB over six and seven generations, respectively. During the final generation of exposure, new dose-response assays and fitness experiments were performed, as described by Belinato et al. [33].

**Table 1:** Effective doses of diflubenzuron (μg/L) for Boa Vista (BVT) and Aparecida de Goiânia (APG), before and after selection with this CSI.

**Pre-selection**							
	EI_50_	EI_80_	EI_95_	RR_50_	RR_80_	RR_95_	slope
Rock A*	0.903	1.251	1.708	1.0	1.0	1.0	5.9
BVT F2	1.119	1.861	3.023	1.2	1.5	1.7	3.8
APG F1	2.104	3.202	4.780	2.3	2.6	2.8	4.6
**Post-selection**							
Rock B*	0.845	1.488	2.555	1.0	1.0	1.0	3.4
BVT F6 cont	1.031	1.656	2.604	1.2	1.1	1.0	4.0
BVT F6 dfb	3.311	4.773	6.768	3.9	3.2	2.6	5.3
APG F7 cont	0.902	2.024	4.381	1.0	1.4	1.7	2.4
APG F7 dfb	3.407	5.779	9.571	4.0	3.9	3.7	3.6

*The Rockefeller strain was used as an experimental control before (Rock A) and after selection (Rock B).

cont: control groups; dfb: groups selected with diflubenzuron.

### Fitness evaluation of the selected mosquitoes

After selection with DFB, several fitness parameters were evaluated. The same measurements were simultaneously carried out using the Rockefeller strain (named 'Rock B' in this work, see above) and the control APG and BVT groups.

### Adult longevity

Three groups of 15 males and 15 females were maintained in small cylindrical cages (8.5 cm diameter and 8.5 cm high), and fed *ad libitum* with 10% sucrose solution, changed twice weekly. The mortality of each gender was scored every two or three days across all groups. Statistical comparisons of survivorship were carried out on the 30^th^ and 40^th^ days post-adult emergence.

### Blood meal acceptance and amount of ingested blood

Three groups of 30 inseminated females, three to five days-old, were maintained in small cages for 24 hours without sugar solution and then fed on an anaesthetised guinea pig, as a blood source, for 30 min. This procedure is approved by CEUA (The Ethics Commission on Animal Use: LW-20/14) of the Oswaldo Cruz Foundation (FIOCRUZ). The number of females that sucessfully fed was recorded, and the amount of blood that they ingested was estimated. Quantification of blood meal size was performed using an analytical balance (APX-200, Denver Instruments). Pools of 10 non-fed and 10 blood-fed females were weighed independently. The mosquitoes in each pool originated from the same experimental group and were reared together. Blood meal size was then calculated by taking the difference between the average weights of the two pools.

### Fecundity and egg viability

Three days after blood feeding, individual females resulting from the assay described above were transferred to inverted Petri dishes, with the lids internally covered by filter paper dampened with 3 mL of dechlorinated water [43]. The plates were kept for 24 hours at 26°C in a BOD incubator, and then the number and the viability of eggs were then recorded.

### Female insemination rate

In order to obtain virgin females, pupae were individually reared in plastic tubes until adult emergence. Adults were sorted by gender and transferred to separate cages. Two to five days after emergence, 15 replicate groups of mosquitoes, each consisting of three females and one male, were transferred to transparent 50 mL Falcon plastic tubes, and fed *ad libitum* with cotton soaked in 10% sugar solution. Three days later, the number of inseminated females in each group was recorded by analysing their spermathecae with the aid of an optic microscope (Nikon Biophot, 200 X). The percentage of males that inseminated 0, 1, 2 or 3 of the females in their group was calculated. The sum of these four percentages was used to evaluate the reproductive capacity of males, by the equation: (∑(♀_0–3_*%♂)). With this formula the resulting numbers could potentially have ranged from zero (if 100% of the males failed to inseminate any females) to 300 (if 100% of the males inseminated each of the three females available).

### Statistical analysis

All bioassays and subsequent fitness assays were repeated at least three times. Data from these independent assays were compared statistically using Student’s *t* or chi-squared tests (χ^2^), as indicated in the results. Longevity data were compared using the Kruskal-Wallis analysis followed by Dunn’s Multiple Comparison for between-treatment analysis. For these comparisons only the p-value is informed in the text. Unless stated, the standard deviation is presented in the text and figures. All statistical analyses were conducted using Graph-Pad Prism version 5.0 for Windows (GraphPad Software, San Diego California USA).

## Results

### Effect of diflubenzuron on *Ae*. *aegypti*

#### Pre-selection

**Fig 1:**
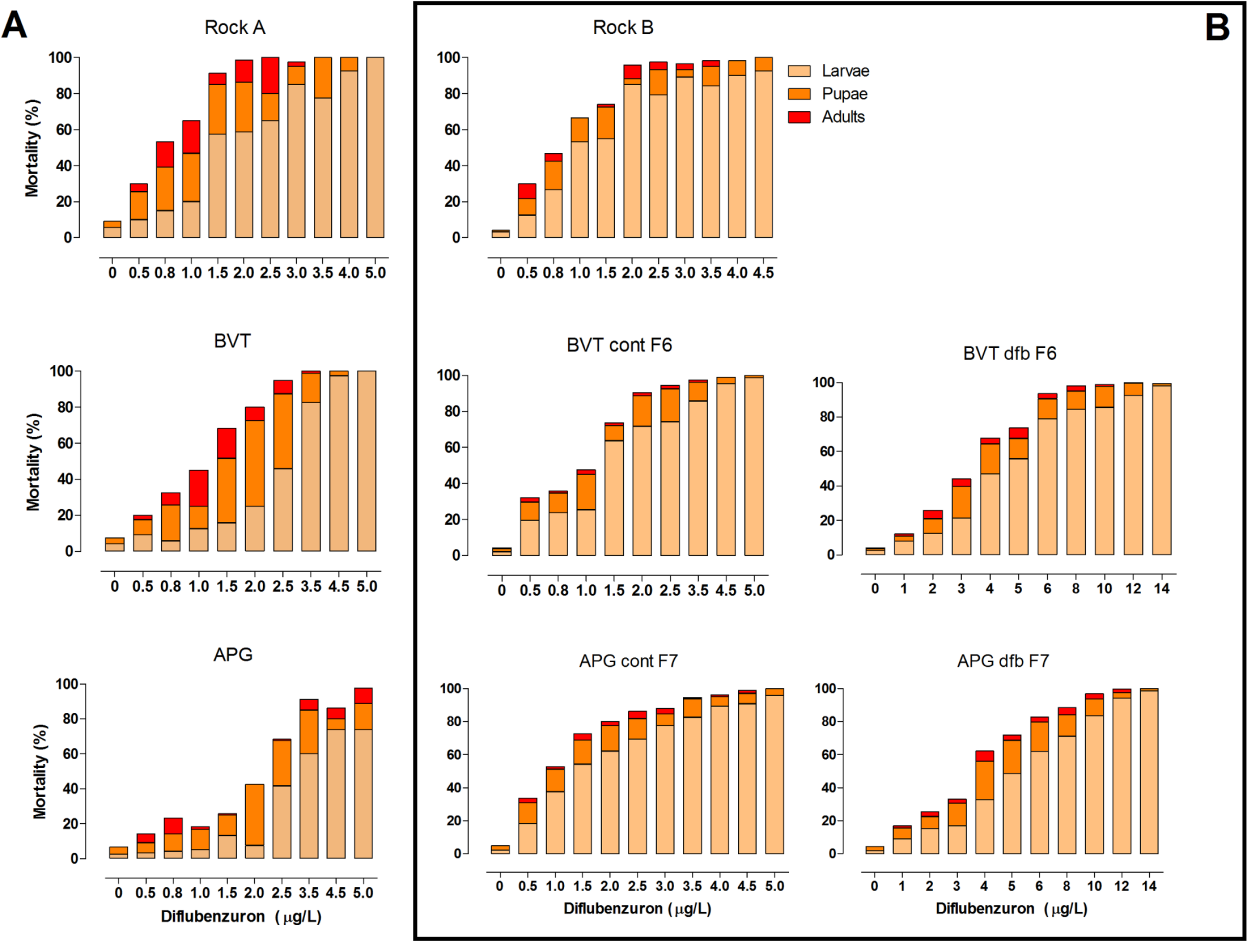
Stage-specific mortality observed for *Ae*. *aegypti* mosquitoes in response to treatment with different concentrations of the insecticide DFB. Mosquitoes from two field-derived Brazilian populations; Boa Vista (BVT) and Aparecida de Goiânia (APG) were compared prior to selection with DFB (Panel A), and then again after the BVT and APG populations had been reared for several generations in the absence (cont) or presence (dfb) of diflubenzuron (Panel B). Note, that in the two rightmost graphs higher concentrations of DFB were used. The selection process lasted for six generations for BVT, and seven for APG. The Rockefeller strain was used as a susceptible control both before (Rock A), and after selection (Rock B).

**Fig 2:**
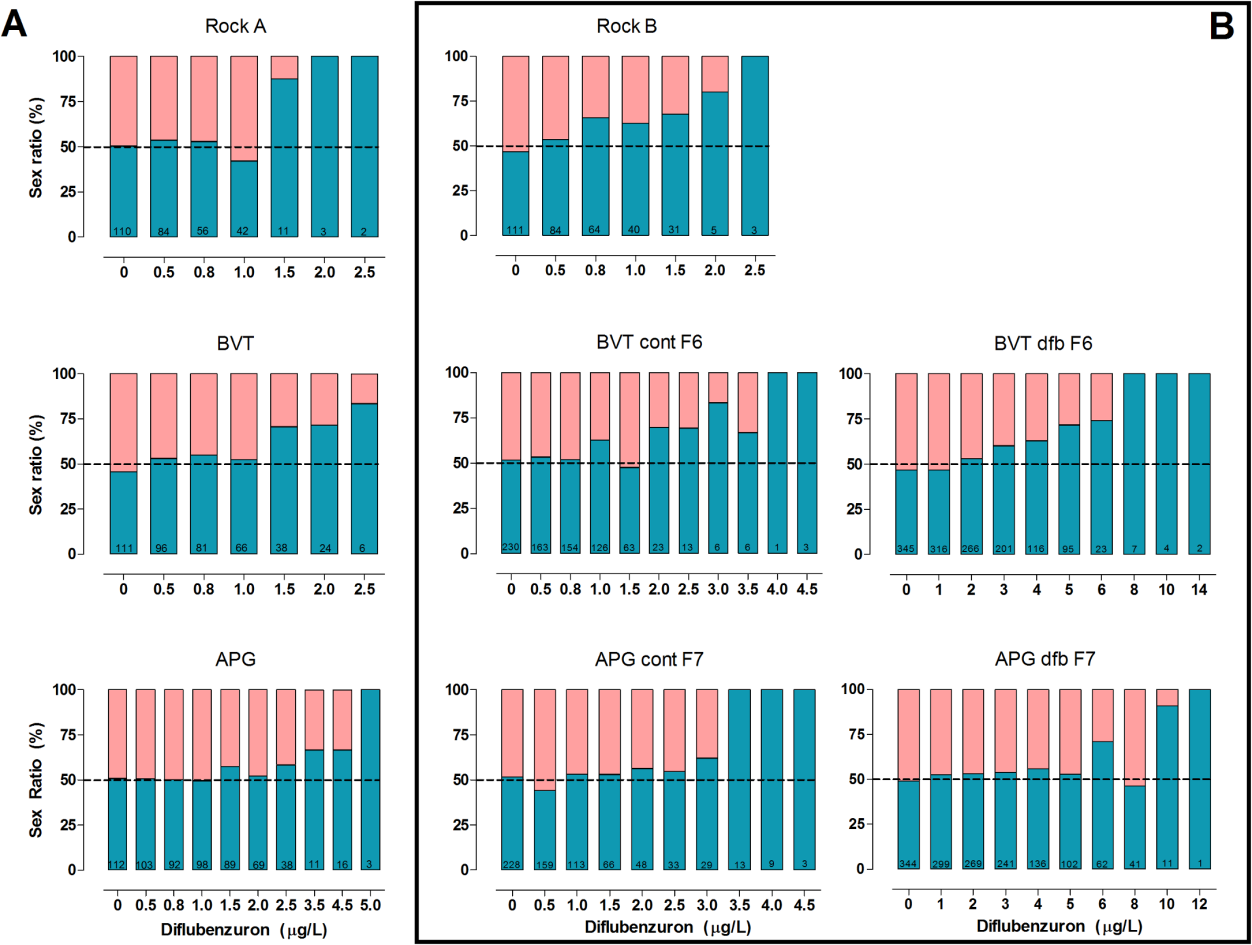
The sex ratio of mosquitoes both pre- (Panel A) and post-selection, (Panel B) showing the proportion of male (blue) and female (pink) adults in each group. The value at the bottom of each bar represents the total number of adults that eclosed in that particular treatment. Diflubenzuron-selected (dfb) and control (cont) strains are shown for the Boa Vista (BVT) and Aparecida de Goiânia (APG) populations.

Dose-response assays confirmed DFB inhibits *Ae*. *aegypti* adult emergence in a dose-dependent manner (S1 Fig). Table 1 depicts the EI values and DFB resistance ratios for both Brazilian populations and the Rockefeller strain, and reveals a higher RR for APG compared to BVT. Evaluation of mortality at each stage shows that the proportion of specimens that died as larvae increased after exposure to higher DFB concentrations. Adult emergence was observed for all CSI concentrations for APG mosquitoes, but not for the three highest concentrations for BVT (Fig 1A). In general, the number of viable adults was very low, and the majority of survivors at high DFB concentrations were males (Fig 2A).

#### Selection with diflubenzuron

**Fig 3:**
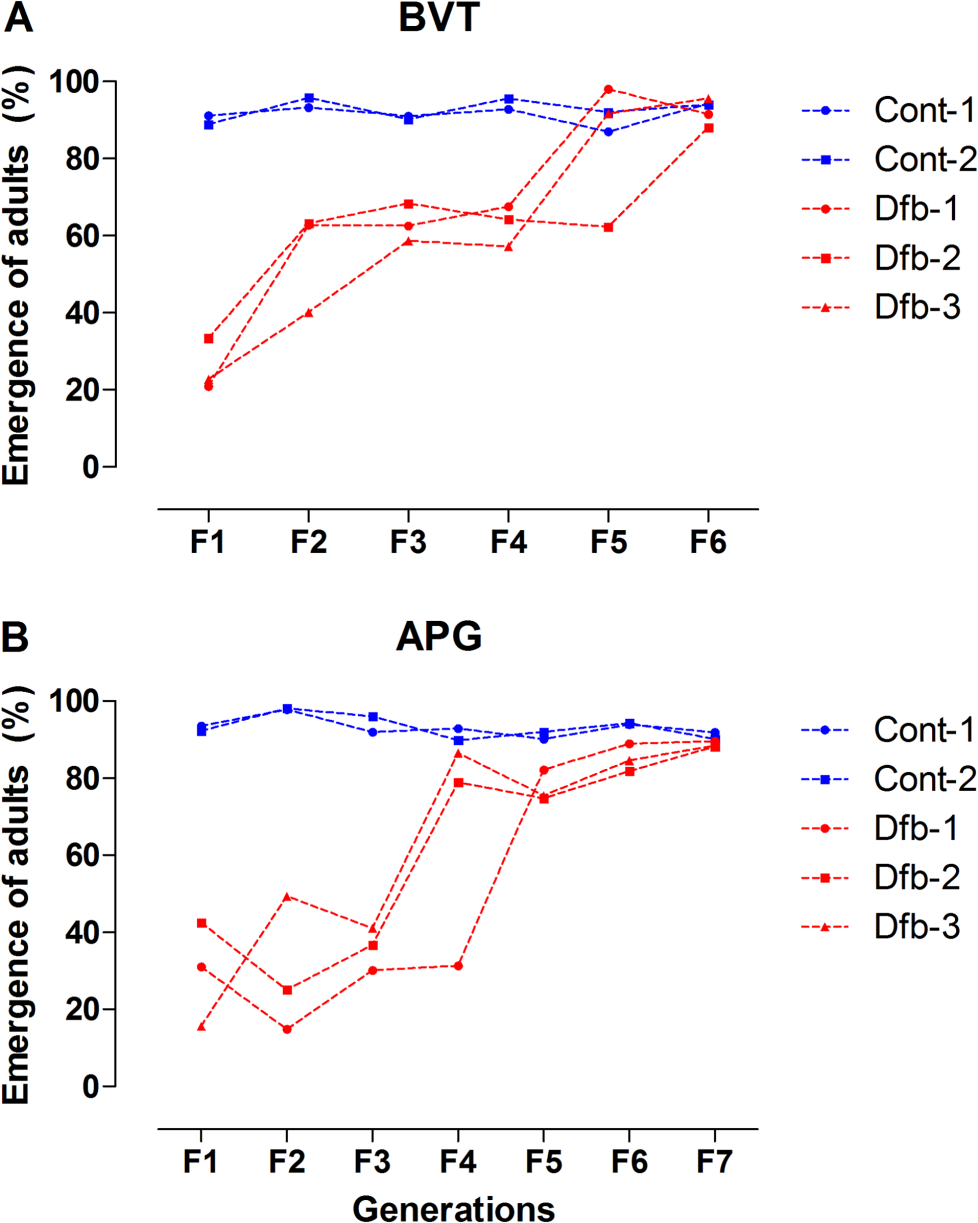
The evolution of DFB resistance in response to successive generations of exposure for BVT (A) and APG (B) mosquitoes, as indicated by an increase in the percentage of mosquitoes surviving to adulthood. The control groups ("cont", in blue) were kept in the laboratory for the same number of generations, and reared without DFB. Curves with the same colors represent replicas.

As stated above, selection of both *Ae*. *aegypti* populations was performed with fixed DFB doses for several generations. These doses (1.8 μg/L for BVT and 3.2 μg/L for APG) corresponded to the IE_80_ observed during the first generation of DFB treatment (Table 1). Although the insecticide pressure was not functionally equivalent throughout the selection process, utilizing a fixed dose better reflects how insecticides are used in the field. Additionally, this method dispenses dose response bioassays at each generation, adding agility to the whole procedure. Simultaneously, for each population, two control groups were maintained without DFB for the same number of generations as the DFB-exposed groups. Although the rate of adult emergence differed slightly between BVT and APG, it increased with each successive generation in both populations, indicating the progression of DFB resistance (Fig 3). For BVT (Fig 3A), the percentage of viable adults had exceeded 50% in two out of three replicates by the F2 generation. While similar proportions of viable adults were only observed for APG in the F4, again in two replicates (Fig 3B). In all cases, adult emergence of the non-treated control groups remained above 85% (Fig 3, blue lines). Furthermore, for each population, at the end of selection, DFB exposed groups displayed adult emergence rates equivalent to the control groups (91.7 ± 3.7% for BVT and 88.7 ± 0.7% for APG). Although there were slight fluctuations across the generations, male/female ratios were generally close to 1:1 (S2 Fig).

#### Post-selection

**Fig 4:**
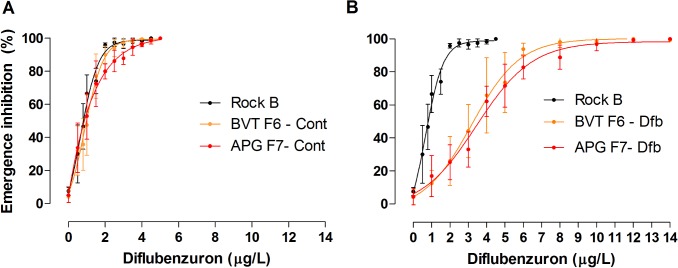
The effect of DFB on adult emergence in the Rockefeller strain (Rock B), control groups (Panel A) and groups selected with DFB (Panel B), during the bioassays conducted post-selection. Curves depict an analysis of non-linear regression (R^2^ > 0.9).

Control and DFB-exposed groups were submitted to DFB dose-response bioassays at the end of the selection process, in order to compare with the initial values (BVT-RR_95_: 1.7; APG-RR_95_: 2.8, see Table 1 for details). In all cases, Rockefeller ('Rock B' in the post-selection fitness assays, see Methods) was used as an insecticide-susceptible control. The effect of DFB remained dose-dependent, similar to what was observed in the pre-selection assays (Fig 4 and S1 Fig). For both the BVT and APG control groups, reared without insecticide, a DFB dose of 5 ug/L was sufficient to completely inhibit adult emergence (Fig 4A). The same concentration, in the DFB-exposed groups, resulted in adult emergence of 15 to 65%. Resistance ratio values were always below 2.0 in the control groups (Table 1, “BVT F6 cont” and “APG F7 cont”). These values corresponded to a susceptibility increase even when compared to the pre-selection populations, APG F1 and BVT F2 (Table 1). In contrast, RR of all exposed groups reached values above 3.0, with the only exception being the BVT RR_95_ (Table 1).

Stage-specific mortality was also evaluated (Fig 1B). As already noted for the pre-selection lines (Fig 1A), in all cases, the rate of early deaths, at the larval stage, increased at higher DFB concentrations. In the control groups, laboratory rearing in the absence of insecticide increased the susceptibility to DFB, as seen by the higher larval mortality compared to the pre-selection assays (compare the two first columns of Fig 1). Conversely, adult emergence was recorded in all post-selection groups treated with DFB doses that had completely inhibited emergence in the pre-selection groups. Across all groups, higher DFB concentrations led to male-biased sex ratios (Fig 2B).

### Analysis of fitness parameters after selection with diflubenzuron

#### Adult longevity

**Fig 5:**
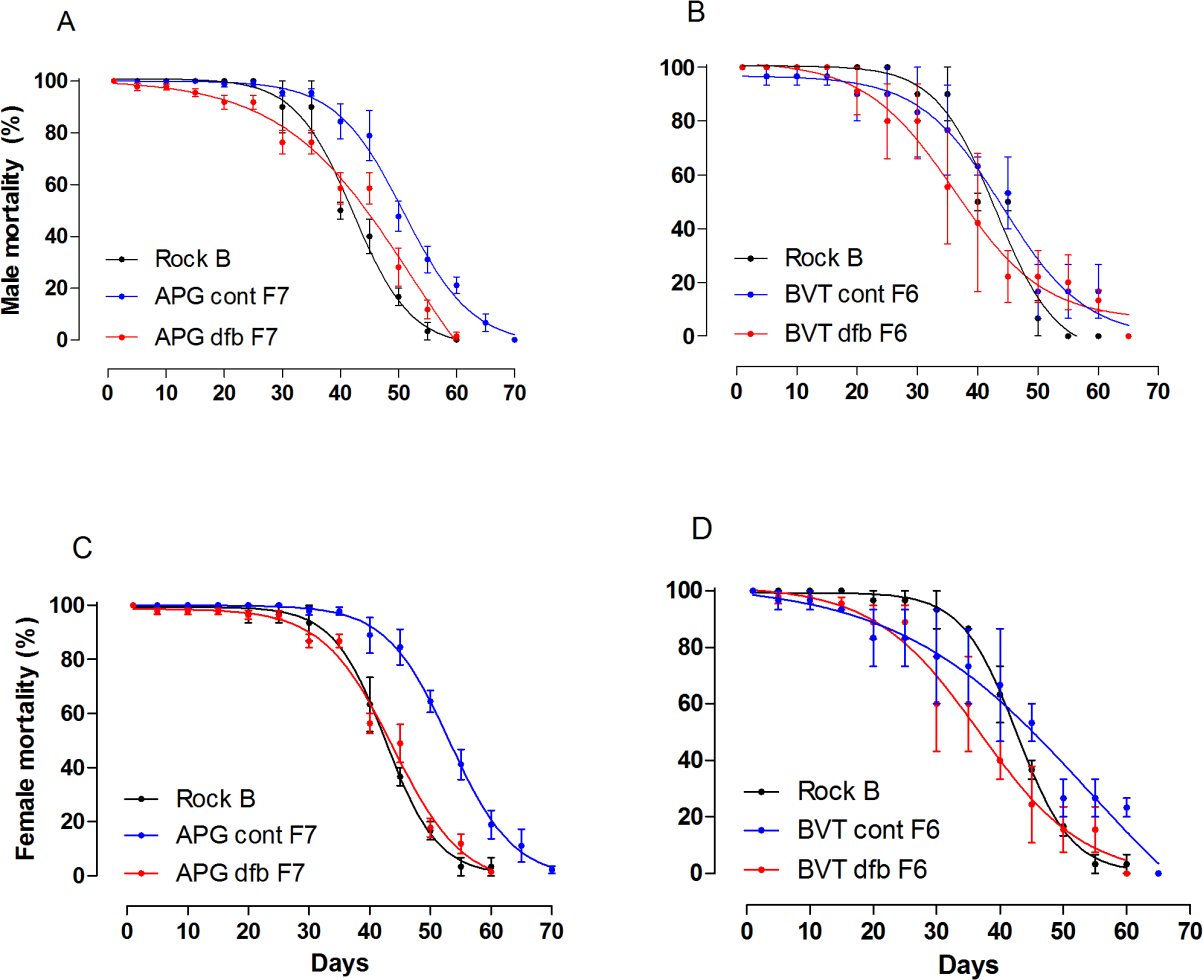
Daily mortality of the Rockefeller strain (black circles), control (blue circles) and selected (red circles) groups of BVT and APG mosquitoes. Panels A and B depict male longevity, while panels C and D show female longevity. All curves depict an analysis of non-linear regression (R^2^ > 0.9).

Daily survival rates of adult male and female mosquitoes are shown in Fig 5. Comparison amongst all groups on the 30^th^ and 40^th^ days after adult emergence revealed no significant differences (Kruskal-Wallis; *P* > 0.05). The only exception was the survival rate of APG control females, higher than both Rockefeller and APG DFB-selected ones (Fig 5C, Kruskal-Wallis; *P* < 0.05).

#### Blood feeding success

**Fig 6:**
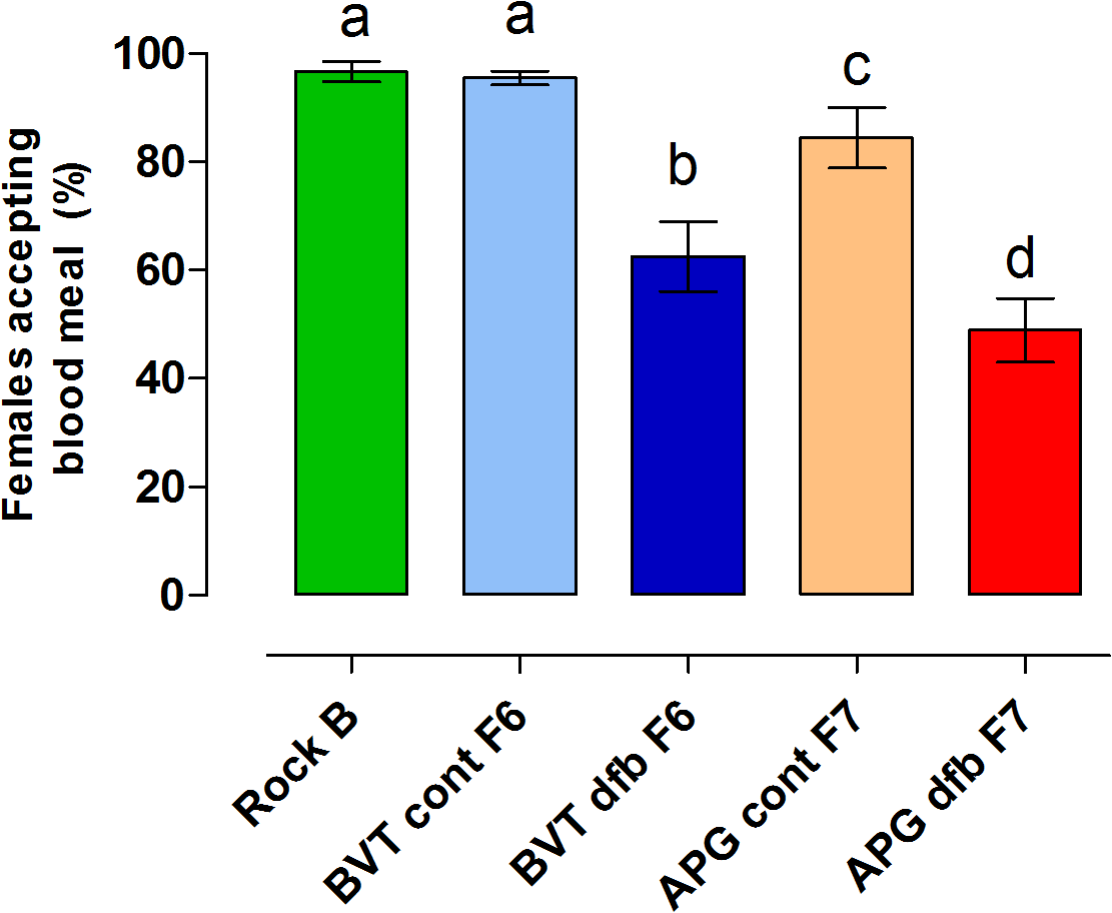
Rate of bloodmeal acceptance for females from the Rockefeller strain (Rock), selected (dfb), or control (cont) groups from Boa Vista (BVT) and Aparecida de Goiânia (APG). Different letters above the columns indicate significant differences among groups (*χ2*; *P* < 0.05).

Approximately 96% (87/90) of the Rockefeller specimens and a similar proportion of BVT control females (95%, 173/181) took a blood meal (χ^*2*^_0.05,1_ = 0,1822*; P* = 0.3347). In contrast, APG control females were less efficient feeders in comparison to the Rockefeller strain (84%, 152/180) (χ^*2*^_0.05,1_ = 13,57*; P* = 0.0001) (Fig 6). Selection with DFB reduced the ability of both populations to successfully obtain a blood meal. Only 62% (168/275) of BVT and 49% (146/270) of APG DFB-selected females ingested blood, and these rates were significantly lower than those observed for the Rockefeller strain (BVT: χ^*2*^_0.05,1_ = 40,76; p < 0.0001; APG: χ^2^_0.05,1_ = 64,67; *P* < 0.0001). For each population, comparison between the control and insecticide-treated groups also shows that selection with DFB significantly impaired blood feeding success (BVT: χ^2^_0.05,1_ = 68,85; p < 0.0001; APG: χ^2^_0.05,1_ = 45,70; *P* < 0.0001). Similarly, there was a significant difference in feeding success between the DFB-selected groups of the two Brazilian populations (χ^2^_0.05,1_ = 8,19; *P* = 0.0021).

#### Amount of ingested blood

**Fig 7:**
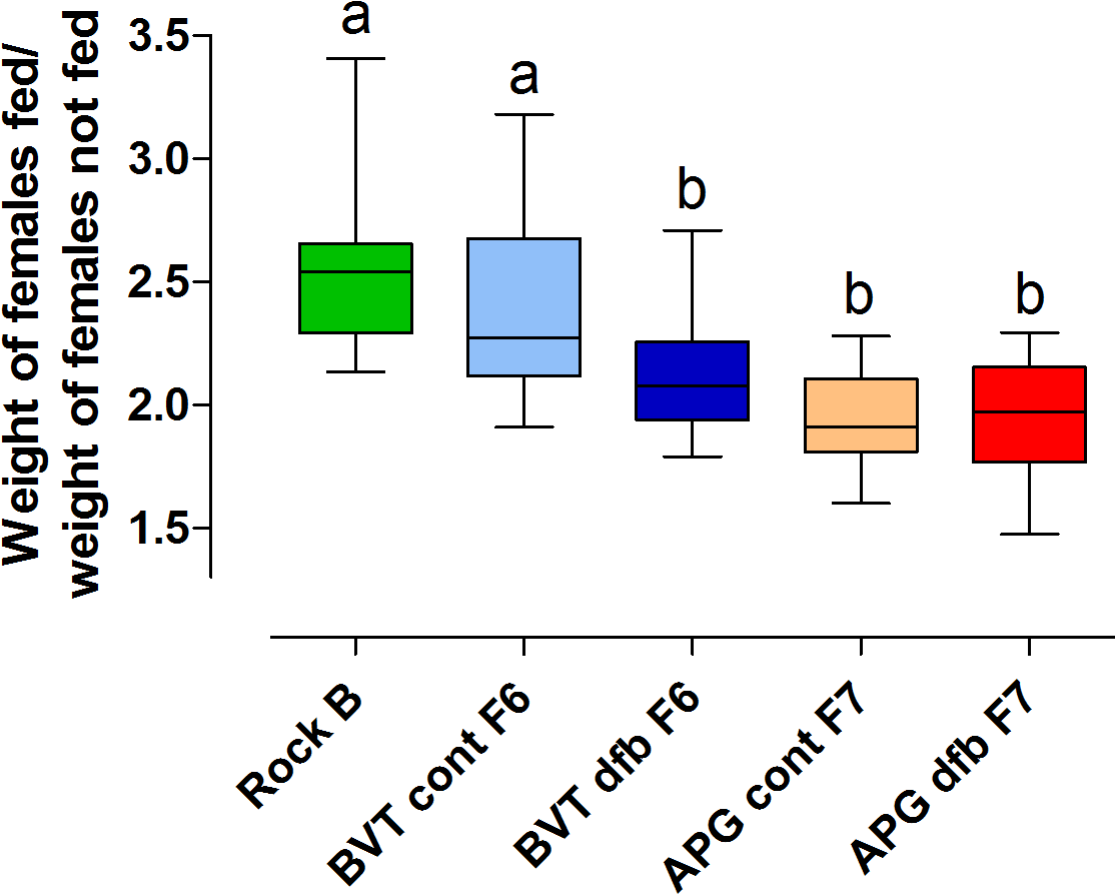
The amount of blood ingested by Rockefeller (Rock) females, and DFB-selected (dfb) or control (cont) mosquitoes from Boa Vista (BVT) and Aparecida de Goiânia (APG). Each box-plot displays the median, the interquartile range and the minimum and maximum amount of blood ingested by each group. Distinct letters indicate significant differences among groups (*t* test*; P* < 0.05).

Rockefeller females ingested around 2.5 ± 0.3 times their weight in blood. A similar amount of blood was ingested by BVT control females (*t*_0.05(1),22_ = 1.119; *P* = 0.1377). In contrast, APG control females ingested 25% less blood than Rockefeller females (*t*_0.05(1),19_ = 4.960; *P* < 0.0001), and 21% less blood than the BVT controls (*t*_0.05(1),27_ = 3.885; p = 0.0003) (Fig 7). BVT females selected with DFB ingested 18% and 11% less blood, respectively than Rockefeller (*t*_0.05(1),21_ = 3.612*; P* = 0.0008) and BVT control females (*t*_0.05(1),29_ = 2.465; *P* = 0.0099). Moderately similar results were observed for APG females selected with DFB, whose volume of ingested blood was 26% lower than Rockefeller females (*t*_0.05(1),18_ = 4.379; *P* = 0.0002). However, control and selected females from this population ingested equivalent amounts of blood (*t*_0.05(1),18_ = 0.1872*; P* = 0.4285). No significant differences were found between the DFB-selected groups from the APG and BVT populations (*t*_0.05(1),25_ = 1.693; p = 0.0515).

#### Fecundity

**Fig 8:**
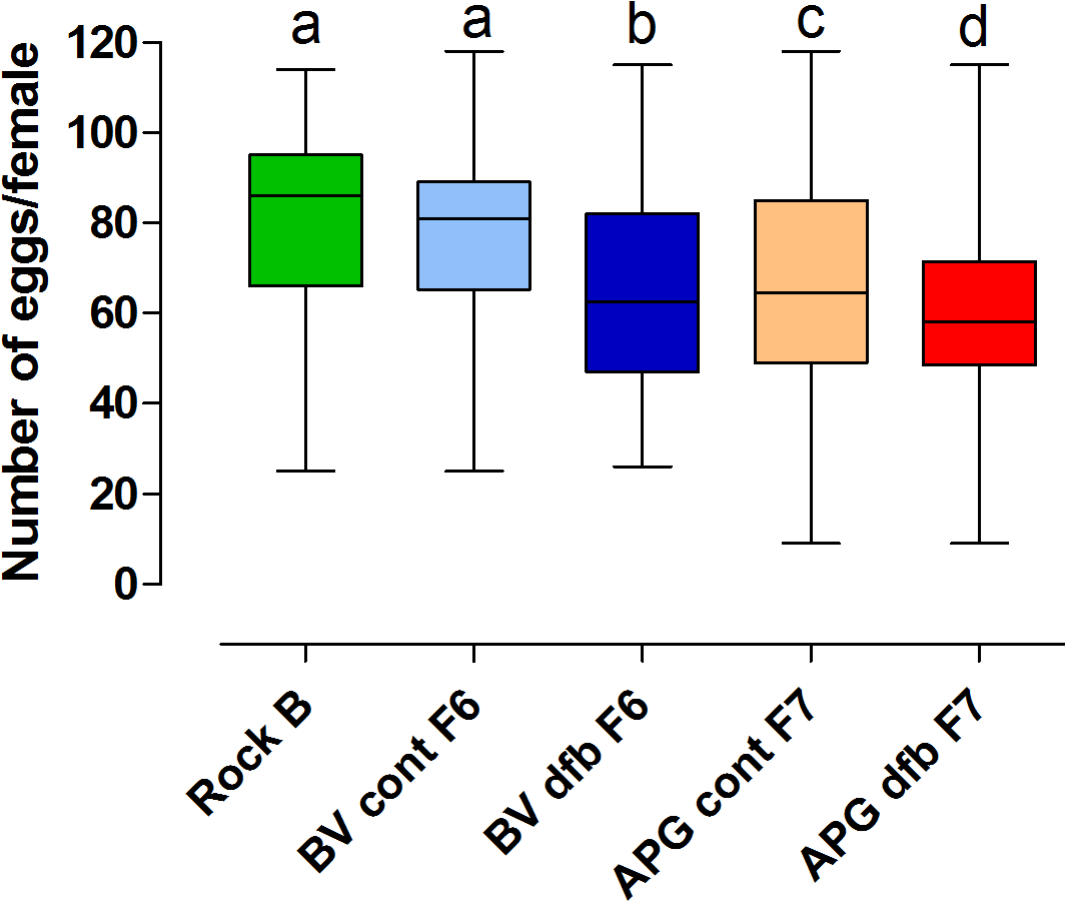
Number of eggs laid by Rockefeller (Rock) females, and selected (dfb) or control (cont) groups of Boa Vista (BVT) and Aparecida de Goiânia (APG) mosquitoes. **Box-plots and letters above them are as for Fig 7**. All groups were compared pairwise except for control and selected groups from each tested population.

Rockefeller females laid an average of 81 ± 19 eggs, which was equivalent to the fecundity levels observed in BVT control females (77 ± 19) (*t*_0.05(1),234_ = 1.642*; P* = 0.0510). While APG control females laid 20% (65 ± 22) fewer eggs than Rockefeller females (*t*_0.05(1),224_ = 5.432; *P* < 0.0001). DFB-treated BVT females laid 21% (64 ± 21) and 16% fewer eggs than Rockefeller (*t*_0.05(1),234_ = 5.930*; P* < 0.0001) and BVT control females (*t*_0.05(1),310_ = 5.410*; P* < 0.0001), respectively (Fig 8). DFB-selected APG females laid 26% (60 ± 18) (*t*_0.05(1),287_ = 8.719*; P* < 0.0001) fewer eggs compared to Rockefeller, and 8% (*t*_0.05(1),353_ = 2.392; p = 0.0086) fewer eggs than APG control females. There were also significant differences in fecundity between the APG and BVT DFB-selected groups (*t*_0.05(1),363_ = 1.964; *P* = 0.0251), and between the control groups of both populations (*t*_0.05(1),300_ = 4.836*; P* < 0.0001).

#### Insemination rate

**Fig 9:**
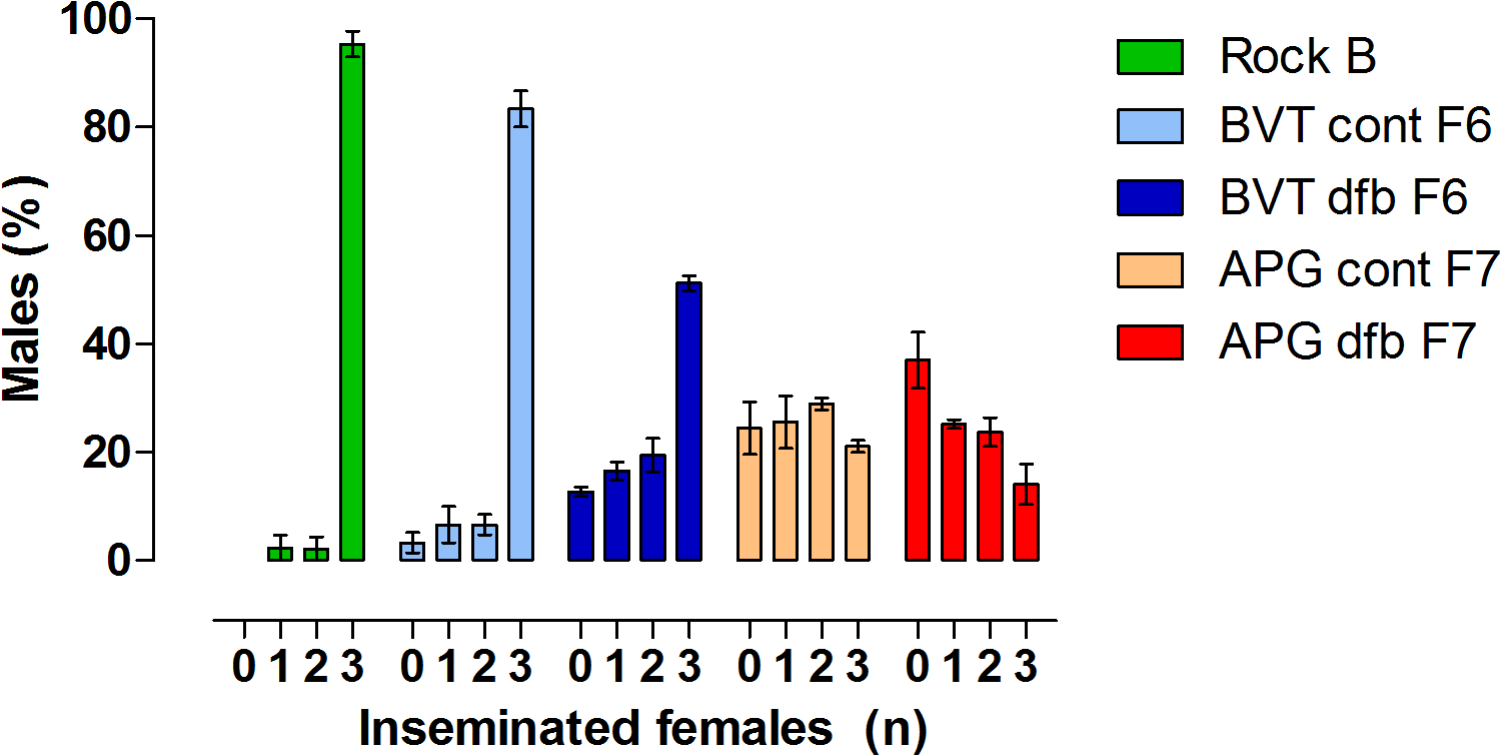
Male mating efficiency for Rockefeller (Rock), and selected (dfb) or control (cont) groups of Boa Vista (BVT) and Aparecida de Goiânia (APG) mosquitoes. For each group, one male was placed in contact with three females for three days. The results are expressed as the percentage of males able to inseminate one, two or three females.

Approximately 97% of the Rockefeller males successfully inseminated the three available females. High insemination rates were also observed in the BVT control groups (83% of males inseminated all females). In contrast, only 50% of DFB-selected BVT males copulated with all three females. In the same group, 12% of males did not mate with any female. Few APG males could inseminate the three females, even in the groups not exposed to DFB. In these control groups only 21% of males copulated with all three females, and after DFB selection this rate dropped to 14% (Fig 9 and S1 Table).

These mating-efficacy data were used to generate an index that compared the male reproductive capacity of the different groups of mosquitoes. This index potentially varies between 0, obtained when no females were inseminated and 300, obtained when all males inseminated all females (see Methods for details). In general, the efficacy of copulation was higher in the control groups of both populations than for mosquitoes from the pre-selection bioassays (Table 2). For instance, the index was 226 for BVT F1 mosquitoes and 270 for the BVT F6 control group. For APG this difference was even more pronounced: with the score of 64 at F1 more than doubled by F7, reaching 146.

**Table 2:** Reproductive capacity of males before and after selection with DFB.

		∑(f0-3*%M)	Reduction (%)
**Preselection** *	Rock A**	297.5	
	BVT F1	226.4	
	APG F1	64.3	
	BVT F1/Rock A		23.9
	APG F1/Rock A		78.4
**Post-selection**	Rock B**	292.6	
	BVT F6 cont	269.7	
	BVT F6 dfb	208.9	
	APG F7 cont	146.4	
	APG F7 dfb	114.5	
	BVT F6 cont/Rock B		7.8
	BVT F6 dfb/Rock B		28.6
	APG F7 cont/Rock B		50.0
	APG F7 dfb/Rock B		60.9
	BVT F6 dfb/cont		29.1
	APG F7 dfb/cont		21.8

*Preselection data were obtained from Belinato et al. [33].

**The Rockefeller strain was used as an experimental control before (Rock A) and after selection (Rock B).

cont: control groups; dfb: groups selected with diflubenzuron.

The efficacy of BVT F1 males, for example, was 24% lower than Rockefeller males. However, in the control group of BVT ('BVT F6 cont') copulatory efficacy was only 8% less than Rockefeller. Similar results were observed for the APG population, where copulatory efficiency was 78% lower than Rockefeller mosquitoes at F1, but only 50% lower at F7 ('APG F7 cont'). Laboratory selection with DFB decreased the reproductive capacity of BVT and APG males by 29 and 22%, respectively, relative to their control groups. However, the selection process did not result in any additional reduction of efficiency compared to Rockefeller males. For BVT, pre-selection males were 24% lower, and post-selection 29% than Rockefeller. While for APG, these values were 78% and 61%, respectively (Table 2).

## Discussion

The search for alternative strategies or products to combat *Ae*. *aegypti*, the vector of dengue virus and other parasites, is a fundamental issue given the ubiquity of resistance against classical neurotoxic insecticides. In Brazil, diflubenzuron, a chitin synthesis inhibitor, currently represents the main compound for the chemical control of *Ae*. *aegypti* larvae that are resistant to the organophosphate temephos. Although several studies have shown the effectiveness of DFB on mosquitoes, knowledge regarding resistance to this CSI is still lacking. We evaluated the efficacy of DFB on two *Ae*. *aegypti* field populations, which were selected for resistance using a fixed dose for several generations. We then evaluated the resistance dynamics and assessed several key fitness parameters of the mosquitoes with and without selection. These results aim to fullfill a knowledge gap relating to this CSI, which was recently highlighted by the WHO as being important to the future control of dengue.

The dose-dependent effect of DFB observed here on *Ae*. *aegypti* was similar to that seen with other CSIs [40,44]. Likewise, the direct relationship between DFB dose and the premature induction of larval mortality seems to be a common effect among CSIs. Novaluron, for example, causes similar effects in *Ae*. *aegypti* and *Cx*. *quinquefasciatus* [44–46], as does triflumuron in *Ae*. *aegypti*, *Ae*. *albopictus* and *Cx*. *quinquefasciatus* [22,40].

Furthermore, the elevated rate of male survival, observed after exposure to high concentrations of DFB, corroborates data obtained for other CSIs [47,48], and is attributed to the faster development of this sex [1] that leads to a shorter period of exposure to the IGR. However, during selection with a fixed dose that corresponded to the IE_80_ for the first generation, a higher proportion of males was only detected for BVT in the first rounds of selection. Sex ratio distortion was never observed for APG. Unexpectedly, we observed that the sex ratio fluctuated only slightly throughout the selection process for either population, and generally remained close to 1:1.

Both the BVT and APG mosquito populations used in these experiments were resistant to temephos [33]. APG presented not only the highest temephos resistance ratio (RR_95_ = 19.2), but also the highest DFB RR, suggesting that there is an association between temephos resistance and tolerance to DFB. A similar effect was observed in *Ae*. *aegypti* exposed to triflumuron [22,40], but not to novaluron [44]. It is possible that tolerance to IGRs is, at least in part, associated with metabolic resistance, a common occurrence in *Ae*. *aegypti* field populations [21]. Diflubenzuron tolerance, for example, has already been associated with increased activity of mixed-function oxidases in blowflies [49].

The aim of the laboratory DFB selection performed here using a single insecticide dose, was to simulate mosquito control in the field. Some differences between the two mosquito populations were noted after the first generations of selection, although their functional significance, if any, is difficult to evaluate. For BVT mosquitoes, the inhibition of adult emergence that was induced by DFB exposure fell to below 50% by the second generation, while for APG mosquitoes, two additional generations were needed to reach this threshold. In contrast, the temephos resistance level of the BVT population (RR_95_ = 7.4) was lower than that of APG (RR_95_ = 19.2).

At the end of the laboratory selection process, an increase in the DFB resistance status was noted in both populations. The opposite situation occurred in the non-exposed control groups, a result that suggests DFB resistance is probably associated with an energy cost potentially affecting the viability and reproduction of mosquitoes. Accordingly, comparison of stage specific mortality with the pre-selection lineage also revealed higher larval mortality levels in the untreated control groups. These data confirm the presence of increased DFB susceptibility in mosquitoes reared in the absence of the insecticide under laboratory conditions for multiple generations.

Several biological traits of both DFB-selected and control groups were evaluated. In order to compare with Rockefeller mosquitoes and with the original field populations [33], Table 3 summarizes the fitness data obtained, except for male insemination efficiency. Whenever a disadvantage was noted for the DFB-selected groups, the percentage reduction, relative to both Rockefeller and the non-DFB exposed counterparts, was calculated.

**Table 3:** Fitness parameters of Boa Vista (BVT) and Aparecida de Goiânia (APG) mosquitoes before (data obtained from Belinato et al. [33]) and after selection with diflubenzuron.

	fitness parameters			
Population/Strain	Longevity^1^	blood meal acceptance^2^	Amount of ingested blood^3^	Number of eggs^4^
				
**Rock A** *	71.1 ± 9.6	95.5 ± 5.0^a^	2.2 ± 0.2^a^	103.2 ± 18.6^a^
**BVT F1**	82.2 ± 16.7	81.1 ± 24.1^b^	2.3 ± 0.1^a^	104.0 ± 17.2^a^
**APG F1**	73.3 ± 13.3	76.6 ± 3.3^b^	1.9 ± 0,1^b^	80.9 ± 29.4^b^
**% reduction:**				
**BVT/Rock A** ^**5**^	_	15.0	_	_
**APG/Rock A** ^**5**^	_	19.7	13.6	21.6
**Rock B** *	63.3 ± 10.0	96.6 ± 3.3^a^	2.5 ± 0.3^a^	81.4 ± 19.5^a^
**BVT F6 cont**	66.6 ± 20.0	95.4 ± 2.9^a^	2.4 ± 0.3^a^	77.0 ± 19.7^a^
**BVT F6 dfb**	40.0 ± 11.5	62.5 ± 19.2^b^	2.1 ± 0.2^b^	64.2 ± 21.9^b^
**APG F7 cont**	88.9 ± 16.1	84.4 ± 13.7^c^	1.9 ± 0.2^b^	65.2 ± 22.3^c^
**APG F7 dfb**	56.3 ± 11.1	48.9 ± 17.7^d^	1.9 ± 0.2^b^	60.0 ± 18.2^d^
**% reduction:**				
**BVTF6 cont/Rock B** ^**5**^	_	1.2	4.0	5.4
**BVT F6 dfb/Rock B** ^**5**^	36.8	35.3	16.0	21.1
**BVT F6 dfb/cont** ^**6**^	39.9	34.4	12.5	16.6
**APG F7 cont/Rock B** ^**5**^	_	12.6	24.0	19.9
**APG F7 dfb/Rock B** ^**5**^	11.0	49.3	24.0	26.2
**APG F7 dfb/cont** ^**6**^	36.6	42.0	0.0	7.9

^1^ percentage of survived females on the 40th day

^2^ percentage of females that accept blood meal

^3^ weight of femals fed/weight of females not fed

^4^ average number of eggs per female

^5^ average reduction in relation to Rockefeller

^6^ average reduction of the selected groups (dfb) in relation to the control groups (cont) of each population

* The Rockefeller strain was used as an experimental control before (Rock A) and after selection (Rock B).

cont: control groups; dfb: groups selected with diflubenzuron.

distinct letters above columns indicate significant differences (*P* < 0.05)

According to Rivero et al. [31], vector longevity is a decisive parameter in the dynamics of disease transmission, as pathogens require a specific period of incubation before being transmitted to a new host. Therefore, the effects of insecticide resistance on longevity can also alter vectorial capacity. Under laboratory conditions, some resistant mosquito populations show reduced longevity when compared with susceptible ones. This is the case for both *Culex pipiens pallens* and *Ae*. *aegypti* after selection for PY resistance [35,50]. Conversely, PY-resistant *Cx*. *quinquefasciatus* females are reported to exhibit increased longevity [51]. As previously shown [33], the longevity of the APG and BVT populations was not significantly affected, even though they were resistant to both OP and PY. Similarly, there was no decrease in longevity observed after selection with DFB, with the only observed change being a longer lifespan for APG control females compared to selected and Rockefeller females (Fig 5). In fact, in the 40th day of adult life, a reduction of at least 35% in the percent of surviving females was noted for both populations in the DFB-exposed groups, related to the control ones (Table 3). However, this difference was revealed to be non-significant. Unless DFB resistance can potentially affect *Ae*. *aegypti* longevity, this effect would be more readily apparent in populations with higher DFB resistance levels.

A higher proportion of DFB-selected females refused to take a blood meal, when compared to Rockefeller or to their respective control groups. The same effect had been previously observed for the original APG and BVT populations (Table 3) [33], and was attributed to temephos resistance. The partial recovery of blood feeding ability observed here for both BVT and APG untreated control groups may be related to the attenuation of temephos resistance that occurred during laboratory adaptation (data not shown) and is probably linked to the reallocation of energy resources in the absence of selection pressure.

Prior to selection the amount of ingested blood and eggs laid by APG females were reduced in relation to Rockefeller and BVT females (Table 3) [33]. These results were primarily attributed to the high temephos resistance levels observed for the APG population. An equivalent situation persisted for the control groups, reared without insecticide in the laboratory. In contrast, all BVT and APG DFB-selected groups exhibited significantly reduced fecundity compared to their respective control groups. In the case of BVT, the observed fecundity decrease was probably related to a reduced blood meal size (compare Figs 7 and 8). However for APG mosquitoes, both control and DFB-exposed groups ingested an equivalent, small-sized blood meal (Fig 7). So consequently this decrease in fecundity indicates a reduced ability to digest blood. This phenotype could have been the result of either the development of DFB selection pressure, or from temephos resistance, which remained at high levels even after DFB selection (data not shown). Although other studies have attributed reduced fecundity to insecticide resistance in mosquitoes [35,50,52,53], they have generally not considered the issue of blood meal size, which is important given the direct link between blood meal size and the number of eggs a mosquito lays [1].

The mating efficacy of the pre-selection populations was lower than for Rockefeller mosquitoes, and was inversely proportional to OP resistance levels [33]. In this work, a partial recovery of this trait was observed in both non-DFB exposed control groups (Table 2), with a stronger effect occurring for the BVT strain. The lesser degree of recovery observed in APG control males was probably related to persisting temephos resistance, as stated above. In contrast, the mating efficiency of DFB-treated males remained low, which suggests it is linked to DFB resistance. Amongst all of the fitness traits evaluated in insecticide-resistant populations, mating success is the least well studied. Berticat et al. [34] showed that insecticide-susceptible *Cx*. *pipiens* males have a mating advantage over competing resistant ones. In combination with our results, this highlights the importance of investigating all facets of reproductive biology when examining resistant populations.

The majority of laboratory studies on fitness in insecticide resistant populations are performed under optimal conditions. Accordingly, the resulting fitness measurements are likely to be underestimated. In the field, mosquito populations are exposed to numerous other deleterious factors beyond insecticides. Therefore, different mechanisms of resistance, or detoxification pathways, could potentially interact or counteract, with the net effect on the evolution of resistance being different and vastly more complicated than what can be observed in the laboratory. In spite of this important caveat, our results confirm that the evolution of DFB resistance results in significant biological costs, in terms of viability and reproductive capacity.

The RRs of *Ae*. *aegypti* to CSIs in all Brazilian mosquito populations are currently below 3.0 [22,40,44]. For the OP temephos, this value is the cut-off point, above which the Ministry of Health has previously recommended interruption of application [21]. One ongoing issue is the absence of field or semi-field studies that show the functional significance of CSI resistance levels. Apparently, *Ae*. *aegypti* populations with an RR of less than 2.0 to the CSI novaluron, are susceptible under field conditions [44]. Simulated field trials with populations that are highly resistant to DFB could prove highly valuable to addressing this potential problem.

Our results indicate that DFB is an effective insecticide against field populations of *Ae*. *aegypti*, including those with high levels of OP resistance. However, laboratory selection with DFB quickly led to the development of resistance in these same mosquitoes, despite associated fitness costs. This highlights the relevance of prudent use of insecticides, and the need for regular monitoring of resistance. We expect these results will prove useful in the design of future mosquito control programs involving CSIs, not only for *Ae*. *aegypti*, but also for other vectors of medical importance.

## Supporting Information

S1 Fig The inhibitory effect of diflubenzuron on adult emergence in Rockefeller strain (Rock A), Boa Vista (BVT) and Aparecida de Goiânia (APG) mosquitoes. The curves represent analysis of non-linear regression obtained during the bioassays (R^2^ > 0.9).
(TIF)

S2 Fig Differences in the sex ratio of the BVT and APG groups in the course of the DFB selection process. The percentage of males is shown in blue while females are in pink.
(TIF)

S1 Table Mating efficacy (%) of Boa Vista (BVT) and Aparecida de Goiânia (APG) males, before and after selection with diflubenzuron. (DOCX)
