# Supplementary figures and images for: The Impact of Selection with Diflubenzuron, a Chitin Synthesis Inhibitor, on the Fitness of Two Brazilian Aedes aegypti Field Populations
Source: PLoS One. 2015 Jun 24;10(6):e0130719. doi: 10.1371/journal.pone.0130719 (PMC4481264; doi:10.1371/journal.pone.0130719)

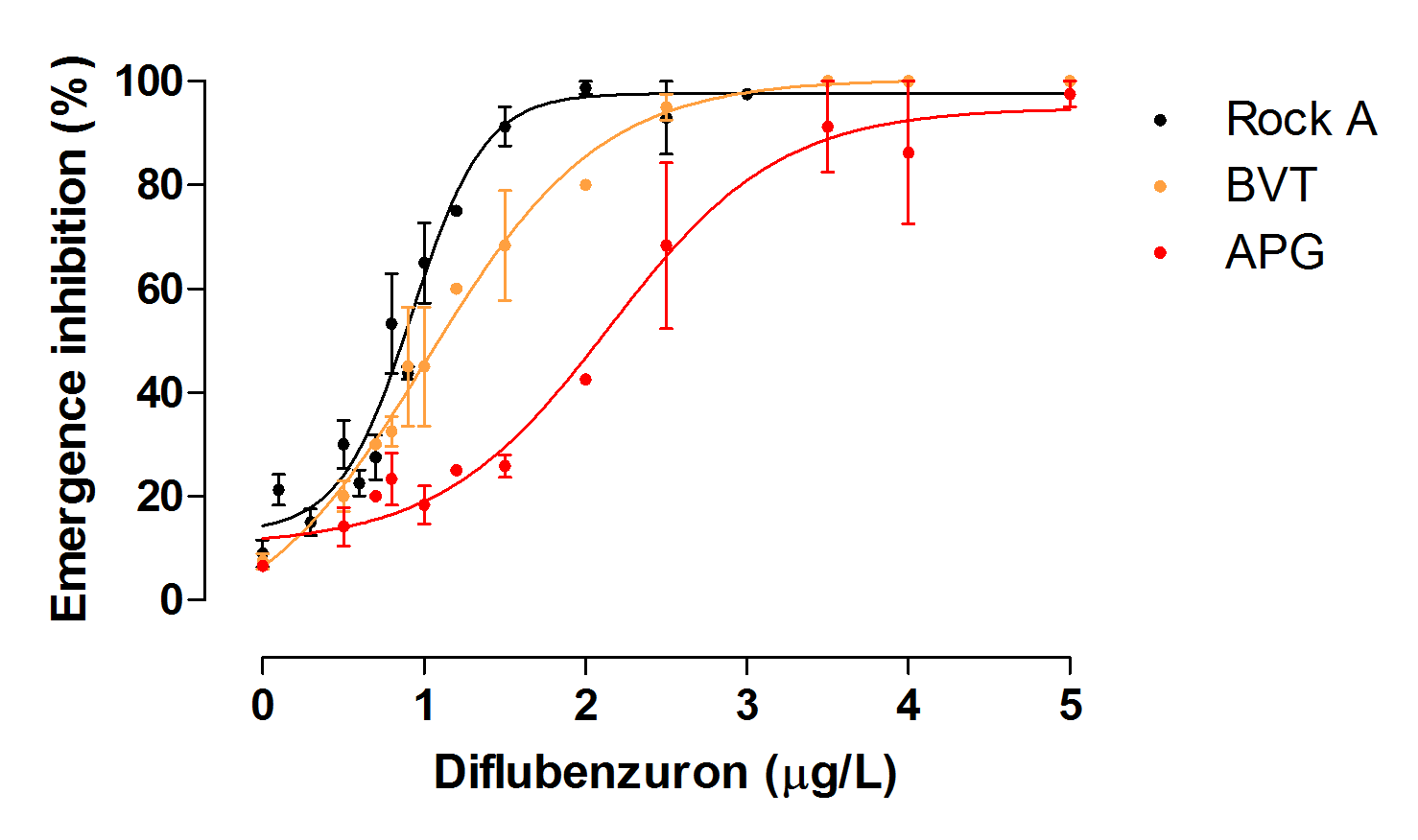

Supplement: S1 Fig — The curves represent analysis of non-linear regression obtained during the bioassays (R2 > 0.9). (TIF) [file pone.0130719.s001.tif]

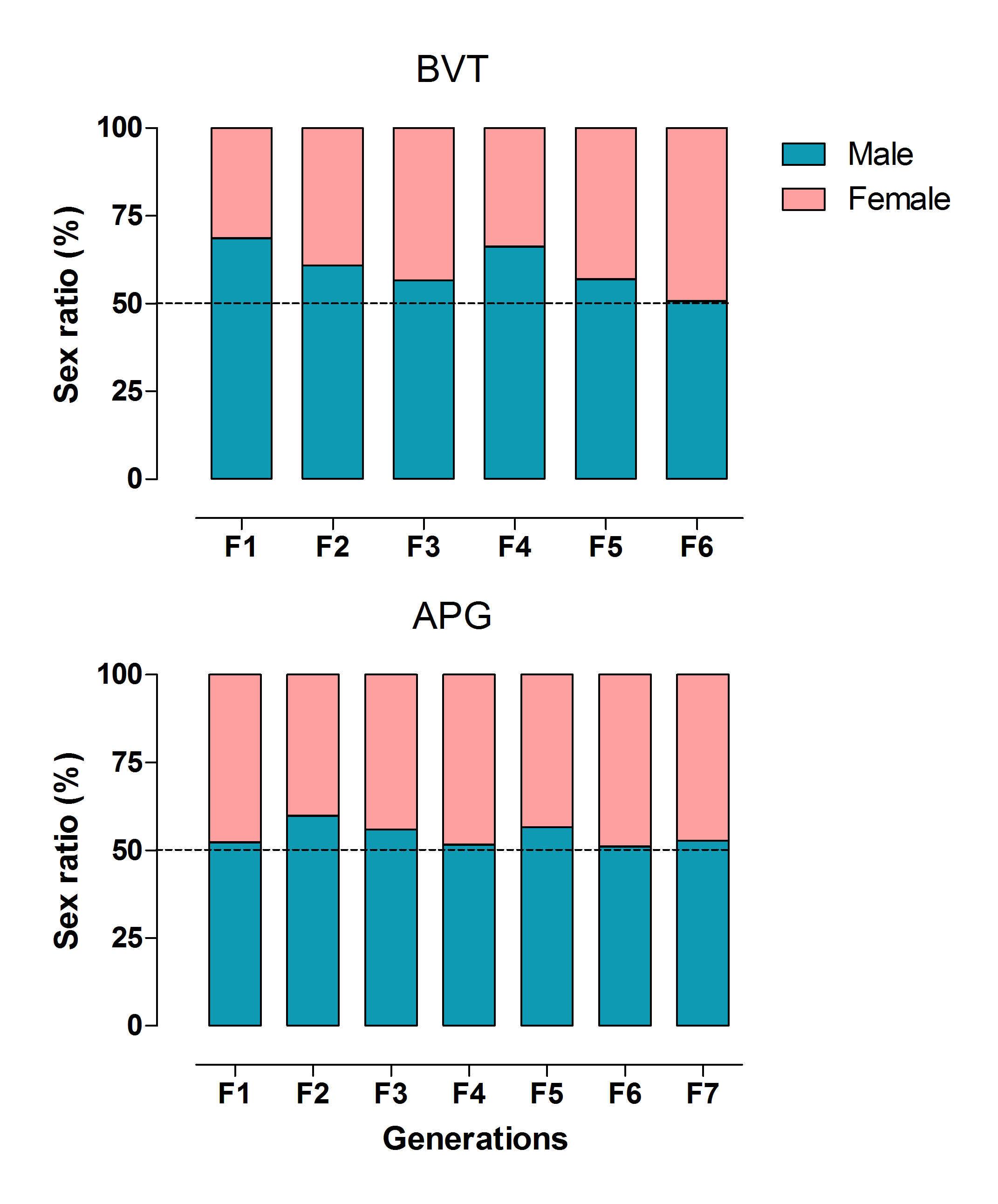

Supplement: S2 Fig — The percentage of males is shown in blue while females are in pink. (TIF) [file pone.0130719.s002.tif]
