# Supplementary material for: The Impact of Selection with Diflubenzuron, a Chitin Synthesis Inhibitor, on the Fitness of Two Brazilian Aedes aegypti Field Populations
Source: PLoS One. 2015 Jun 24;10(6):e0130719. doi: 10.1371/journal.pone.0130719 (PMC4481264; doi:10.1371/journal.pone.0130719)
Supplement: S1 Table — (DOCX) [file pone.0130719.s003.docx]

| Preselection* |  |  |  |  |  |
| --- | --- | --- | --- | --- | --- |
| Inseminated females (n) | Rock A** | BVT F1 | APG F1 |  |  |
| 0 | 0 | 6.6 ± 6.7 | 57.7 ± 10.2 |  |  |
| 1 | 0 | 13.3 ± 6.7 | 26.6 ± 6.7 |  |  |
| 2 | 2.2 ± 3.8 | 26.6 ± 11.5 | 8.8 ± 3.8 |  |  |
| 3 | 97.7 ± 3.8 | 53.3 ± 6.7 | 6.7 ± 6.7 |  |  |
| Post-selection** |  |  |  |  |  |
| Inseminated females (n) | Rock B** | BVT F6  cont | BVT F6  dfb | APG F7  cont | APG F7  dfb |
| 0 | 0.0 | 3.3 ± 3.3 | 12.7 ± 1.4 | 24.4 ± 8,4 | 37.0 ± 9.0 |
| 1 | 2.3 ± 4.1 | 6.6 ± 5.8 | 16.5 ± 2.9 | 25.5 ± 8.4 | 25.1 ± 1.3 |
| 2 | 2.2 ± 3.8 | 6.6 ± 3.3 | 19.4 ± 5.2 | 28.8 ± 1.9 | 23.7 ± 4.6 |
| 3 | 95.3 ± 4.0 | 83.3 ± 5.8 | 51.2 ± 2.4 | 21.1 ± 1.9 | 14.0 ± 6.4 |

*Data from Belinato et al. [33].

**The Rockefeller strain was used as an experimental control before (Rock A) and after selection (Rock B).

cont: control groups; dfb: groups selected with diflubenzuron.
